# Oculomotor Abnormalities and Nystagmus in Brainstem Disease: A Mini Review

**DOI:** 10.3390/audiolres15060150

**Published:** 2025-11-06

**Authors:** Augusto Pietro Casani, Mauro Gufoni, Nicola Ducci, Giacinto Asprella Libonati, Giuseppe Chiarella

**Affiliations:** 1 ENT Section, Medical, Molecular and Critical Area, Department of Surgical Pathology, Pisa University Hospital, 56122 Pisa, Italy; mgufoni@gmail.com (M.G.); 29550320@studenti.unipi.it (N.D.); 2 Department of Otolaryngology and Cervico-Facial Surgery, Madonna delle Grazie Hospital, 75100 Matera, Italy; giacintoasprella@gmail.com; 3 Unit of Audiology, Regional Centre of Cochlear Implants and ENT Diseases, Department of Experimental and Clinical Medicine, Magna Graecia University, 88100 Catanzaro, Italy; chiarella@unicz.it

**Keywords:** brainstem, vertigo, nystagmus, ocular motor, vestibular central disorders, eye movements

## Abstract

The brainstem plays a pivotal role in the generation and control of eye movements—including saccades, smooth pursuit, the vestibulo-ocular reflex (VOR), vergence, and gaze holding. Beyond its vital physiological functions, it is also essential for the coordination of balance and movement. Consequently, eye movement disorders of brainstem origin are often accompanied by vertigo, imbalance, unsteady gait, and diplopia, particularly during changes in head or body position. A sound understanding of the neural structures involved in oculomotor and vestibular control is therefore crucial for accurately identifying and localizing a wide variety of brainstem syndromes. However, oculomotor abnormalities resulting from brainstem disease represent a major diagnostic challenge for the neurotologist, owing to the wide spectrum of possible etiologies (vascular, traumatic, degenerative, neoplastic), their variable severity and clinical course (acute, fluctuating, or progressive), and the frequent concomitant involvement of other central structures, particularly the cerebellum. This mini review summarizes the pathophysiological mechanisms and clinical features of oculomotor disorders and nystagmus associated with brainstem disease.

## 1. Introduction

The term “central vestibular dysfunction” covers a wide range of pathologies, ranging from a single acute event such as a cerebellar stroke to chronic and fluctuating conditions such as vestibular migraine. The spectrum of the diseases causing dizziness and vertigo is quite complex, crossing multiple disciplines (neurology, otorhinolaryngology, internal medicine, etc.): at present, the classification method is based on the anatomical site involved, and the nature of the disease is mainly used for the etiological diagnosis of dizziness/vertigo. However, in some cases, the localization and possible etiological diagnosis are difficult, and the real incidence of central vestibular disorders is reported differently, ranging from 13.3 to 17.6% [1,2,3], also depending on the setting of the study (single-center, multi-center, primary care, emergency room, neurology, or otolaryngology department).

A central vestibular disorder can be defined as a condition originating from lesions in the brain, cerebellum, and brainstem. Evaluating and treating all these pathological conditions originating from different brain sectors represents an extremely complex challenge for the neurotologist by virtue of the remarkable diversity of causes (vascular, traumatic, degenerative, neoplastic) of their variable severity and based on the progressive nature of some diagnoses of central vestibular pathology. Going into specifics, the brainstem represents a structure of prominent importance for the generation and control of eye movements (saccades, smooth pursuit, the vestibulo-ocular reflex (VOR), vergence, and gaze holding) [4]. In addition to its several physiological functions of vital importance, the brainstem plays a role in the coordination of balance and movements. This process is based upon the activity of the oculo-vestibular pathway system. This system consists of a vestibular part, which controls the body position, and its activity is coordinated with that of the regulatory neurons, which regulate the postural skeletal muscles and the eye movements. Therefore, eye movement disorders of brainstem origin are frequently associated with vertigo, imbalance, unsteady gait and walk, and diplopia upon changing head and body posture. For these reasons, an accurate characterization of the eye movement abnormalities represents a fundamental moment in the diagnosis of central vestibular syndromes, being also able to identify and localize, at least in a high number of cases, the role of each unit and/or neural circuit in the genesis of the oculomotor lesion (Table 1) [5]. The clinical examination of the oculomotor and vestibular system can be integrated by an instrumental examination including the caloric test and the video head impulse test, the latter allowing an evaluation of the vertical canal too [6,7]. Additionally, the investigation of postural stability in patients suffering from central vestibular disorders can be achieved using different methods such as ultrasound-computer-craniocorpography [8].

## 2. Symptoms and Signs in Brainstem Lesions

The central vestibular disorders manifest with nonspecific symptoms (dizziness and vertigo). Whether we are faced with a central or peripheral problem, the symptoms are few and not localized to a particular area and include the following:

- rotatory vertigo;
- postural instability or unsteadiness;
- postural crises;
- unclear or blurred vision.

**Figure 1:**
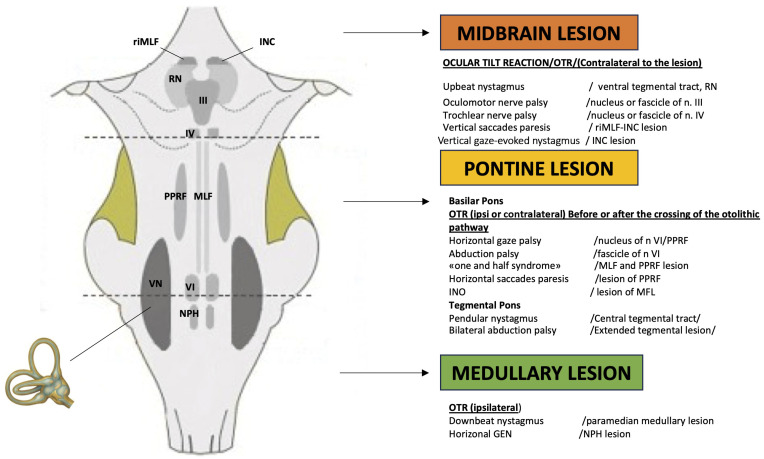
Schematic illustration of brainstem structures involved in ocular motor control and relative consequences in case of lesion. The vestibular end organ is schematically represented on the left side. The continuous black line represents the vestibular nerve connecting the inner ear to the vestibular nuclei (VN). INC: interstitial nucleus of Cajal; MLF: medial longitudinal fascicle; NPH: nucleus propositus hypoglossi; PPRF: paramedian pontine reticular formation; riMLF: rostral interstitial nucleus of the Medial Longitudinal Fasciculus; RN: nucleus of Roller; III: oculomotor nerve; IV: trochlear nerve; VI: abducens nerve.

However, none of these symptoms are indicative of the site of the lesion: rotatory vertigo is typical of an acute disorder of the peripheral vestibular receptor but is also present in Wallenberg syndrome or in other vascular disorders related to the brainstem or cerebellum. At the same time, chronic instability or dizziness with no rotatory vertigo can be due to a neoplastic or degenerative disease of the central nervous system as well as to a bilateral peripheral vestibular dysfunction, uncompensated unilateral vestibular loss, or a late stage of Ménière’s disease. Lateropulsion and subsequent risk of fall, characterized by a sudden onset and rapid resolution, can be present in the neurovascular conflict of the eighth cranial nerve or in patients with vertebrobasilar insufficiency, as well as in Tumarkin’s otolithic crises in late Ménière’s disease [9]. For this reason, it is not possible to attempt a diagnosis based only on the type of vertigo (“objective” or “subjective”, or as proposed more recently, without substantial changes in meaning, “external” or “internal”). Unlike subjective symptoms, objective symptoms can be highly specific for location, and the oto-neurological examination can provide the additional sign that, demonstrating a spatial–temporal dissemination, allows the neurologist to make a diagnosis in case of vertebrobasilar insufficiency or multiple sclerosis. Many brainstem centers are involved in controlling eye movements, but a simple clinical rule can be used in clinical practice: horizontal eye movements are generated and controlled in the pontine region, whereas vertical (and torsional) eye movements originate in the midbrain (Figure 1); consequently, the recognition of the various patterns of oculomotor disturbances could allow a topographic diagnosis of the lesions occurring at diverse levels in the brainstem. Classically, symptoms and signs of brainstem involvement are classified as medullary, pontine, and mesencephalic syndromes [10,11]. In the next paragraphs, the oculomotor abnormalities of greatest neuro-otological interest will be reported.

### 2.1. Abnormal Eye Movements in Medullary Lesions

**Figure 2:**
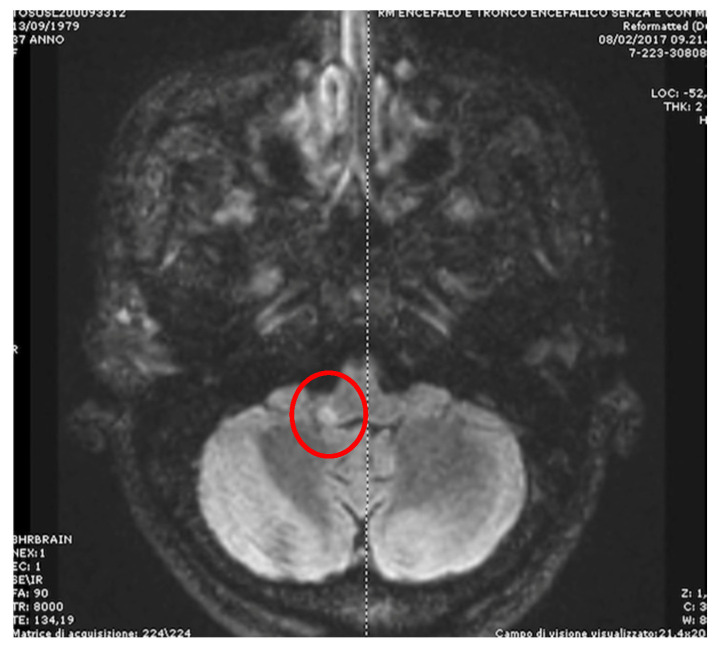
**A** female, 46 years old, complaining of acute spinning vertigo with no other accompanying symptom, presented to the emergency room: The bedside examination reveals pure torsional nystagmus (see Supplementary Material); MRI (T2-weighted axial images with contrast) shows a demyelinating focal disorder localized in the right posterior medulla at the level of the inferior cerebellar peduncle (red circle).

A lot of medullary structures are involved in the control of eye movements (vestibular nuclei, nucleus propositus hypoglossi (NPH), nucleus intercalatus, nucleus of Roller, inferior olivary nucleus, inferior cerebellar peduncle, and cell groups of the paramedian tract). A lesion of one or more of these areas could induce various patterns of nystagmus and vertigo. Acute rotatory vertigo with spontaneous torsional or horizontal rotary unidirectional nystagmus could be the initial symptomatology of Wallenberg’s syndrome (lateral medullary infarction), often mimicking a vestibular neuritis [12]. The presence of some additional signs (alterations in facial sensation, dysarthria and dysphagia, and ipsilateral Horner’s syndrome) could help reach a correct diagnosis. However, the combination of ocular lateropulsion and saccadic dysmetria with a partial or complete ipsilesional ocular tilt reaction (OTR) is indicative of a lateral medullary lesion [13,14]. On the contrary, a medial medullary infarction, in addition to a contralateral hemiparesis and ipsilesional tongue paralysis, generates an ipsilateral horizontal (sometimes upbeating for involvement of perihypoglossal nuclei) nystagmus and a horizontal gaze-evoked nystagmus (GEN) more intense-looking to the affected side (caused by damage to the NPH) [15]. Unilateral lesions of NPH induce intense vertigo and unsteadiness associated with ipsilesional-beating spontaneous nystagmus or horizontal GEN more intense on looking toward the ipsilesional side, central patterns of head-shaking nystagmus (HSN), impaired smooth pursuit (SP), and static contralateral ocular deviation [16]. Furthermore, patients with NPH lesions showed decreased head impulse VOR gains for the contralesional horizontal semicircular canal and increased gains for both anterior semicircular canals [17]. The inferior cerebellar peduncle, via the juxtarestiform body, allows a direct connection between the vestibular nuclei and the cerebellum, and a lesion involving this structure induces acute vertigo associated with ipsilesional spontaneous nystagmus (horizontal and/or torsional) and contralateral ocular tilt reaction (OTR), caused by a loss of cerebellar inhibition of the vestibular nuclei [18,19] (Figure 2). A lesion of the nucleus of Roller could generate an upbeat nystagmus just as a lesion of the cell groups of the paramedian tracts can generate a downbeat or upbeat nystagmus, this structure being involved in the processing of the vertical position of the eyes [20,21,22].

### 2.2. Abnormal Eye Movements in Pontine Lesions

**Figure 3:**
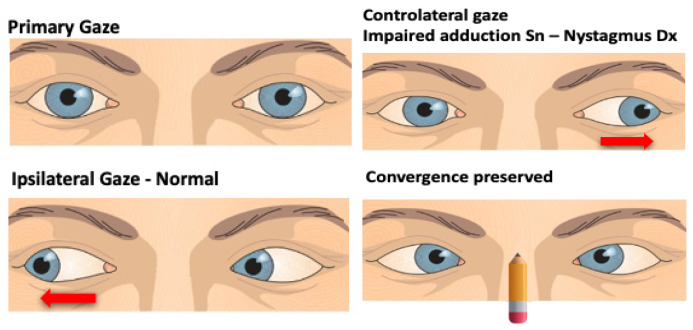
Right internuclear ophthalmoplegia. Primary gaze: no ocular abnormalities. Ipsilateral gaze: normal. Contralateral gaze: no adduction in the right eye and abducting nystagmus in the left eye. The convergence is bilaterally preserved.

The pontine level is located at the center for horizontal saccades, the paramedian pontine reticular formation (PPRF); this structure, together with the NPH, the vestibular nuclei, and the vestibulo-cerebellum, represents the center for the conjugate horizontal gaze-holding function, the so-called “neuronal integrator.” Clinically, this means that isolated horizontal saccadic palsy indicates a pontine lesion, and a unilateral PPRF (or sixth nerve nucleus) lesion will result in saccadic disturbances on the side of the lesion. Isolated palsy of horizontal saccades on both sides was found in spinocerebellar ataxia type 2 or brainstem tumor [23,24]. A purely horizontal GEN originates from a pontine lesion [4]. On the other hand, a vertical GEN and impaired vertical smooth pursuit and saccades may appear because of an involvement of the pontine areas connected by the medial longitudinal fascicule (MLF) to the midbrain area involved with vertical gaze [25]. Another key structure at the pontine level is the medial longitudinal fascicle (MLF), whose damage causes the onset of the so-called internuclear ophthalmoplegia (INO), caused by an interruption of the MLF between the nuclei of the third cranial nerve on one side and the contralateral sixth. The INO can be unilateral or bilateral and may present with or without (neurologically isolated) other brainstem findings. Unilateral INO (caused mainly by ischemia) is characterized by impaired adduction of the ipsilateral eye and abducting nystagmus (dissociated nystagmus) of the contralateral eye, sometimes associated with skew deviation (SD) characterized by hypertropia of the ipsilesional eye and contraversive head tilt [4,26,27] (Figure 3). Bilateral INO is characterized by bilateral adduction lag and bilateral abduction nystagmus, associated with spontaneous (upbeating) and gaze-evoked vertical nystagmus [4,22,28], and the most common etiology is infarction of the midbrain in older patients and demyelinating disease in young patients [26,29,30]. However, an MLF lesion could cause vertical (upbeating, more in the contralesional eye, and downbeating, more in the ipsilesional eye) or torsional nystagmus, each of which may cause oscillopsia (illusion or “feeling” of unstable vision in which a person perceives that their environment is moving) [4,13]. A lesion involving the PPRF and the MLF induces “one and a half syndrome.” This syndrome is characterized by the loss of all horizontal movements except abduction in the contralateral eye, so the only preserved horizontal eye movement is abduction of the contralateral eye [31,32]. This condition is frequently associated with an involvement of one or more cranial nerves [33].

### 2.3. Abnormal Eye Movements in Midbrain Lesions

The midbrain contains the third and fourth nerve nuclei, the rostral interstitial nucleus of the medial longitudinal fasciculus (riMLF), and the interstitial nucleus of Cajal (INC). These structures represent the center involved in vertical saccades and in vertical gaze-holding function [34]. Clinically, a midbrain lesion induces an isolated vertical saccadic paresis and/or isolated vertical GEN [35,36,37,38]. However, it must be emphasized that a lot of the lesions affecting the brainstem, in different pathologies (degenerative, neoplastic, vascular), lead not only to the onset of oculomotor abnormalities and/or nystagmus. The midbrain, pons, and medulla are components of the brainstem that control basic body functions such as consciousness, breathing, proprioception, heart rate, and blood pressure; for this reason, a lesion of these structures also leads to other neurological symptoms such as headache, sensory disturbances, and altered function of some cranial nerves (Table 2), often making the above-described abnormalities a part (albeit a significant one) of a more complex neurological clinical picture. In this context, it is necessary to emphasize the role of a correct evaluation of the cochlear symptoms using a battery of audiological testing, even if some of them (i.e., auditory evoked potentials) usually are not available in acute settings. Nonetheless, an adequate knowledge of the neural structures involved in oculomotor and vestibular control allows for the identification and accurate localization of a wide variety of brainstem syndromes.

## 3. Vascular Disorders of the Brainstem

Posterior circulation strokes involving the brainstem can result in subsequent oculomotor and vestibular disorders. Approximately 20–25% of ischemic strokes involve the posterior circulation [39]. The brainstem is supplied by the vertebrobasilar system, which includes the following arteries: vertebral arteries, basilar arteries, posterior inferior cerebellar artery (PICA), anterior inferior cerebellar artery (AICA), superior cerebellar artery, and labyrinthine artery. Strokes involving the arteries supplying the brainstem can result in a variety of different neurologic manifestations—such as ocular motor palsies, lid retraction, ptosis, diplopia, gaze palsies, nystagmus, SD, issues with accommodation, and abnormal pupillary function. Among the possible causes of acute vertigo, disorders of the posterior circulation constitute one of the most common central forms. Approximately 10% to 20% of patients with acute vertigo presenting to the emergency department have had a stroke [40,41], particularly involving the brainstem or, more frequently, the cerebellum, 95% ischemic [9] as a cause. While the most common manifestations of posterior fossa infarction are vertigo and/or dizziness, in these cases other cerebellar and/or brainstem symptoms or signs usually accompany these symptoms. Nevertheless, focal cerebellar stroke, especially in the PICA area of vascularization, may mimic acute unilateral peripheral damage, with acute vertigo being the only presenting symptom [42,43]. Neurologic exams in stroke patients presenting vertigo or dizziness are non-focal in >80% of cases, even when performed by an experienced neurology-trained neuro-otologist, and even when the highest-risk-for-stroke population is studied [44]. Furthermore, even when focal signs occur, they are often subtle, especially for frontline clinicians.

For these reasons, in the most recent studies, the authors have concentrated a lot, and with some success, on the differential diagnosis between a peripheral and central cause of acute vertigo: in the last years, methods and tests have been proposed to allow the differential diagnosis [41,45,46,47], even if with a sensitivity that does not reach one hundred percent [48]. On the other hand, the coexistence of other neurological signs and symptoms is crucial for the diagnosis of acute vertigo of central origin. Nevertheless, differential diagnosis between an acute vertigo of peripheral origin and brainstem or cerebellar infarction may be challenging, as focal strokes affecting the root entry zone of the eighth nerve, the vestibular nuclei, or the pathways (in the cerebellar peduncle) from the vestibular nucleus to the cerebellar flocculus and vermis could mimic a peripheral acute vertigo due to a vestibular neuritis. In these cases, in the absence of signs or symptoms clearly attributable to the central nervous system involvement, an accurate bedside vestibular examination could be more sensitive in the diagnosis of an ischemic event than MRI in the first 24–48 h [47]. Non-contrast computed tomography [NCCT] is the most commonly employed neuroimaging modality in the ED for patients presenting with neurological symptoms, including acute vertigo. Despite its rapid accessibility, its diagnostic yield is limited, ranging from 2 to 10% [49]. In cases of isolated acute vertigo without other indicative case history or clinical findings of cerebral pathology, NCCT has been shown to lack additional diagnostic value, discouraging its routine use [50]. Clinicians should be aware of the test’s low sensitivity, where a negative result may be misleading. Some diagnostic algorithms based on easy bedside tests have been proposed to avoid misdiagnosis in an emergency setting, such as HINTS (assessing HIT, direction of spontaneous nystagmus, and SD), HINTS plus (HINTS + evaluation of hearing loss), and STANDING (assessing direction of spontaneous and positional nystagmus, HIT, and gait) [45,47,48,50,51,52]. In fact, multidirectional (gaze-evoked) or mainly vertical/torsional spontaneous nystagmus, a negative HIT, a significant SD, and severe unsteadiness should be addressed towards a central origin and should prompt further investigations. In particular, a negative HIT stands as a pivotal and often decisive clinical examination along with the presence of a gaze-evoked nystagmus (a bidirectional nystagmus induced by moving the eyes to the eccentric position left and right, with fast phases beating in the direction of gaze). Another hallmark sign of the peripheral origin of acute vertigo is the fixation suppression of spontaneous nystagmus: nystagmus is enhanced or brought out by removing fixation with Frenzel glasses or, better, with infrared video Frenzel goggles. However, unlike cerebellar infarction, which can manifest itself in a significant percentage of cases with a clinical picture very similar to acute peripheral vertigo [43], brainstem infarction is much more frequently associated with neurological signs. As already mentioned above, a lateral medullary stroke from an involvement of PICA (Wallenberg syndrome) can result in acute vertigo and imbalance with horizontal–torsional spontaneous nystagmus (with fixation, the fast phase is directed toward the intact side, while with loss of fixation, the patients’ eyes deviated tonically toward the affected side), ipsilesional OTR, hypermetric saccades to the side of the lesion, hypometric to the other side accompanied by classic neurological signs, including ipsilateral loss of pain and temperature sensation of the face (involvement of the descending nucleus and tract of the trigeminal nerve), contralateral loss of pain and temperature sensation in the trunk and limbs (spinothalamic tract), dysphagia (nucleus ambiguous), ipsilateral Horner syndrome (descending oculo-sympathetic tract), ipsilateral limb ataxia, gait ataxia, and lateropulsion (inferior cerebellar peduncle, ICP) (Table 2) [10,13,53]. A pontine ischemia induces a disruption of the horizontal gaze pathway, resulting in sixth nerve palsy, INO, horizontal gaze palsies, or a combination of these pathologies, as in one and a half syndrome. These abnormalities and nystagmus can be associated with hemiparesis, central facial nerve palsy, hemisensory loss, dysarthria, dysphagia, ataxia, and pseudobulbar affect [54]. Finally, an infarction restricted to the vestibular nuclei may present with acute isolated vertigo with features of both peripheral and central vestibulopathies: spontaneous torsional–horizontal nystagmus that beats away from the lesion side, direction-changing gaze-evoked nystagmus, and positive HIT [55]. Accordingly, central signs, including the HINTS and “Standing”, should be carefully sought even in patients with features of peripheral vestibulopathy and negative MRI.

## 4. Neuro-Otological Signs Associated with Brainstem Involvement

Until a few years ago, it was believed that the pathology of the brainstem could not be monosymptomatic: the brainstem is so rich in structures involved not only in postural and oculomotor control but also in maintaining consciousness, in controlling the sleep–wake cycle and the respiratory and cardiovascular activities, and in the control of sensory inputs. By virtue of this extreme functional complexity, it seems logical to hypothesize that a lesion (vascular, neoplastic, degenerative, etc.) can generate numerous symptoms and signs of central origin. However, there are many cases in which the lesions of the brainstem can determine exclusively neurotological signs.

### 4.1. Central Positional Nystagmus

Central positional nystagmus (CPN) can be caused by cerebellar and/or brainstem disorders involving the central vestibulo-cerebellar pathways. These include, among others, structural lesions (tumors, stroke, multiple sclerosis), autoimmune (paraneoplastic and non-paraneoplastic) degenerative disorders, and genetic ataxias [56]. CPN could mimic benign paroxysmal positional vertigo (BPPV), especially certain atypical and rarer forms of BPPV presenting with positional downbeat nystagmus, with upbeat nystagmus when the patient returns to the sitting position, or with nystagmus that does not fatigue either in BPPV of the posterior semicircular canal or in the apogeotropic type of BPPV of the horizontal semicircular canal [57]. BPPV is typically caused by migration of degenerated otoconia into the semicircular canals, rendering them sensitive to head motion and inducing a nystagmus compatible with the involvement of the posterior semicircular canal (torsional geotropic with an upbeating component) or lateral semicircular canal (bidirectional horizontal, geotropic, or apogeotropic). CPN can appear as persistent (nystagmus present in one or more positions of the head which, in the single position, does not present any changes for as long as it lasts, whether it is short or long-lasting) or transitory or sometimes paroxysmal, even if the two forms can coexist or follow one another, as in the case of a paroxysmal CPN which, maintaining the lying position, becomes persistent [58,59]. CPN can be associated with intense vertigo, oscillopsia, and neurovegetative symptoms, particularly the paroxysmal form.

CPN is probably caused by an abnormal integration of signals coming from semicircular canals and involving the cerebellar nodulus and uvula, inducing a disinhibition of irregular afferent signals converging on the vestibular nucleus [60]. The same cerebellar structures play a key role in the mathematical integration of signals from otolithic and semicircular canal input so that a graviceptive variation induced by the change in position could be the trigger for CPN in case of nodulus/uvula dysfunction [61]. For these reasons, CPN is more common in cerebellar disease, and brainstem lesions were the cause of CPN only in 8.5% of all the cases [62]. In identifying the site of the lesion, it must be kept in mind that the inferior cerebellar peduncle originates anatomically from the brainstem but is functionally part of the cerebellum [63]. Features of central positional nystagmus include the following [58,63,64,65]:

- The CPN may have any trajectory, but pure downbeat and apogeotropic bidirectional horizontal forms are far more common than upbeat, torsional, or mixed forms.
- Nystagmus that occurs during or shortly after a change in position, with little or no latency, suggests a central cause.
- Failure to fatigue/persistence of nystagmus, especially after repeated supine roll tests, suggests a central cause.
- Intense positional nystagmus with little to no vertiginous sensation may also suggest a central cause.
- Poor or no response to repeated repositioning maneuvers.

**Figure 4:**
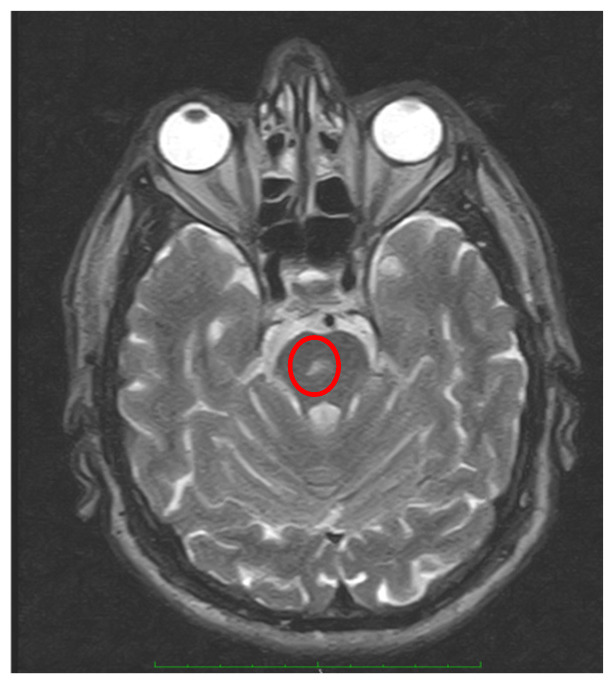
Male, 72, showing at bedside examination a positional bidirectional apogeotropic nystagmus (see Supplementary Material) not responding to repeated repositioning maneuvers. Axial MRI images (T2-weighted with contrast) show a small vascular lesion located at the medial/paramedial right center pontine level (red circle).

Obviously, in contrast to BPPV of peripheral otolithic origin, in the large majority of patients with CPN, additional neurological or ocular motor symptoms or signs are typically present (GEN, saccadic smooth pursuit, central patterns of head-shaking nystagmus, abnormalities of saccades, ataxia, and other signs of brainstem dysfunction) [62,64]. The most common patterns of CPN are the following:

- Apogeotropic bidirectional horizontal nystagmus. More commonly associated with cerebellar disease [56,64], this type of CPN shows no latency and no associated vertigo, lasts as long as the position is maintained, and is reproduced by returning the patient to the same position. A brainstem lesion could induce an apogeotropic CPN because of damage to the connection from the nodulus and uvula (and sometimes tonsil) to the vestibular nuclei [18,58,59,60,61,62,63,64] (Figure 4).

- Positional downbeating nystagmus (PDN). In the past, the presence of PDN during the head-hanging position and/or in Dix–Hallpike was considered a sign of central vestibular involvement; in the present time, PDN is more frequently associated with an apogeotropic variant of posterior canal BPPV [65] or anterior canal BPPV [66]. Two patterns of PDN can be recognized: paroxysmal, with poor or no latency, duration less than 1 min, and occasionally with an upbeating nystagmus when the patient returns to the sitting position; and persistent, sometimes preceded by a paroxysmal component [67]. The pathophysiology of PDN during a brainstem lesion is similar to that described for the apogeotropic horizontal positional nystagmus. Recently a case of paroxysmal CPN mimicking posterior canal BPPV due to a pontine infarction was described [68]. Finally, upbeating nystagmus and central bidirectional geotropic nystagmus of central origin are much rarer.

### 4.2. Head-Shaking Nystagmus (HSN)

The head-shaking test (HST) consists of a series of 10 to-and-fro motions in the horizontal plane performed by the examiner on a patient wearing Frenzel glasses or infrared video Frenzel goggles. In a normal subject at the end of the test, no nystagmus is detected. HST may elicit or enhance nystagmus in patients with vestibular imbalance, suggesting a peripheral vestibular lesion. After a head-shaking test is performed on the horizontal plane, a downbeat (more frequently), upbeat, or torsional nystagmus appears or clearly superimposes over a concomitant horizontal spontaneous nystagmus, and a central pattern of HSN (the so-called perverted HSN) is evident [69,70,71]. Another central pattern of HSN is represented by the appearance of HSN after very few (2–3) cycles of passive oscillation of the head performed at a very low speed, a stimulus not capable of generating a “quantity” of energy sufficient to load the velocity storage mechanism [69,70,71,72]. The presence of HSN in central vestibular disorders has been revisited in patients with Wallenberg syndrome, where HSN was observed in 14 of 16 patients, and, in all cases, the horizontal component was ipsilesional. Even in the eight patients with contralesional spontaneous horizontal nystagmus, the HSN was opposite to the spontaneous nystagmus. Three patients showed unusually strong HSN with a maximum slow-phase velocity greater than 60 degrees/second. MRI demonstrated in all the cases a lateral medullary infarction involving the caudal and middle part of vestibular nuclei with sparing of the rostral part [72]. A perverted HSN was described in caudal medullary lesions [70,73,74] and in pontine lesions [70,74]. In 23 cases of vertebrobasilar stroke, 17% of atypical HSNs were detected; only in 1 case did the MRI show a medullary infarction with a direction of HSN opposite to spontaneous nystagmus associated with evident ataxia [75].

#### Smooth Pursuit and Saccades Abnormalities in Brainstem Lesions

**Smooth pursuit (SP)** is induced by retinal slip with the purpose of keeping the image of a slowly moving object stable within the fovea along with a role in suppressing or reinforcing the VOR during visual stimulation. Therefore, in case of cerebellar or brainstem lesions, smooth pursuit, optokinetic reflex, and visual control of the VOR usually show the same degree of impairment. In lateral medullary syndrome, impaired smooth pursuit toward the opposite side of the lesion is observed [13,14]. A brainstem lesion at the caudal level can cause a reduction in the gain of the contralateral direct smooth pursuit, while the ipsilateral slow movements are preserved [76]. In twelve patients with ventromedial pontine infarction, seven had pursuit with saccadic substitutions, but only two of them had vertigo [77]. An asymmetric alteration of pursuit, which is normal in one direction and low gain in the other, may be suggestive of a cerebellar or brainstem lesion, while the bilateral and symmetric alteration is nonspecific for location [76,77]. Other regions in the brainstem may cause abnormalities in smooth pursuit if infarcted, but smooth pursuit deficits are commonly overshadowed by other more significant neurologic deficits.

**Saccades** are responsible for rapidly moving the gaze from one fixation point to another; abnormalities of accuracy, latency, and velocity of saccades, such as intrusion and saccadic oscillation, can occur involuntarily in pathological conditions [4]. The riMLF and PPRF burst neurons affect the velocity and initiation of saccades in vertical and horizontal directions, respectively. This phenomenon can be caused by lesions in various structures such as the cerebellum, brainstem, basal ganglia, and cerebral hemispheres, and it prevents the gaze from staying in one place. A decrease in the speed or absence of saccadic movements in the horizontal plane is instead typical of the brainstem, due to lesions affecting the MLF or the PPRF [13,14,23,24,25]. Hypermetric saccades to the side of the lesion and hypometric to the other side are typically encountered in Wallenberg syndrome, and this pattern of saccadic abnormalities associated with ocular ipsipulsion is highly localizing for this syndrome [5,35,53]. The far less common medial medullary lesion could produce an opposite pattern: ipsilesional saccadic hypometria and contralesional hypermetric saccades, sometimes associated with ocular lateropulsion [15]. In milder cases of INO, patients may only demonstrate slowed adducting saccades, so-called adduction lag [78]. As already described, a midbrain lesion generates an isolated vertical saccadic paresis sometimes associated with isolated vertical GEN [35,36,37]. A combination of ipsilesional hypometric saccades, contralesional saccadic smooth pursuit, and unilateral gaze-evoked nystagmus has been considered as typical of a focal brainstem (pontine) lesion [79].

### 4.3. Ocular Tilt Reaction (OTR)

The otolithic pathways that modulate the VOR originate from the utricular and saccular maculae and project to the ipsilateral vestibular nuclei at the pontine–medullary junction. This pathway then decussates to the contralateral side at the pontine level to reach the pons and midbrain in the MLF until it reaches the supranuclear centers for vertical–torsional eye movements in the rostral midbrain (riMLF and INC, whose activity not only generates vertical and torsional saccades but also acts as the neural integrator for vertical and torsional gaze-holding) [80,81]. A lesion along this pathway (from the peripheral organ to the supratentorial structures) induces the so-called ocular tilt reaction (OTR), consisting of SD, ocular torsion, head tilt, and deviation of the subjective visual vertical, all tilted toward the lower (hypotropic) eye [80]:

- Skew deviation is a vertical misalignment of the eyes due to unilateral impairment of the otolith–ocular reflex. Hypotropia of the eye (on the side of the lesion if the damage affects the peripheral receptor and/or the pathways before their crossing, contralaterally in case of deficit after the commissure).
- Ocular torsion (in the case of the right labyrinth, counterclockwise torsion from the viewer’s point of view in the case of a pre-decussation lesion, clockwise in the case of a post-decussation lesion).
- Head tilt (to the side of the lesion if the damage affects the peripheral receptor and/or the pathways before their crossing, contralaterally in case of deficit after the commissure).

**Figure 5:**
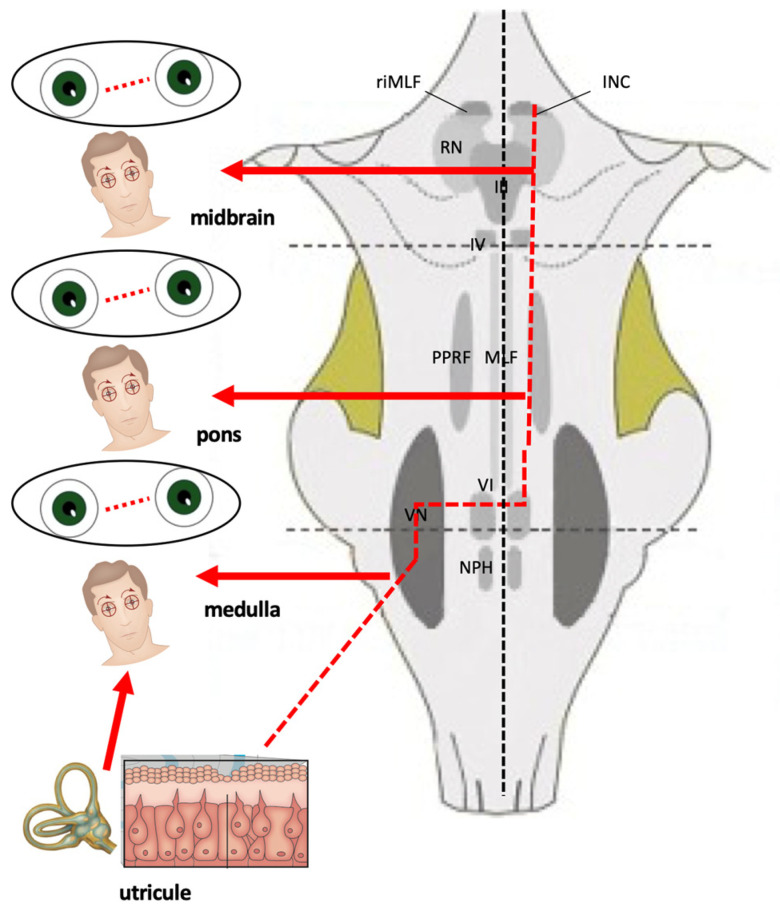
Depending on the location of the lesion (before or after decussation), the side of the hypotropic eye may or may not coincide with the side of the lesion.

The pathway for the otolithic ocular responses decussates in the pons; hence, static ocular tilt reactions from hypofunction are ipsiversive (lower eye on the side of the lesion) with peripheral vestibular and pontomedullary lesions and contraversive with ponto-mesencephalic lesions [80,81,82] (Figure 5). Whereas most cases of OTR are tonic and due to a decrease in tonic neural activity, paroxysmal OTR is due to intermittent unilateral hyperfunction, with tilt in the direction opposite to that of tonic ocular tilt reactions. A paroxysmal OTR was described in cases of upper brainstem lesions [83,84]. In the presence of acute spontaneous vertigo, evaluating SD in a bedside examination as part of the HINTS paradigm is a crucial sign for differential diagnosis between a peripheral or central vestibular disease: usually large-amplitude SD points to a central lesion [85,86]. For this latter purpose, when spontaneous horizontal nystagmus is associated with OTR, a correlation between nystagmus direction and SD could provide additional useful information. If the nystagmus has the fast phase away from the hypertropic eye (called UPHILL nystagmus), this could be considered a peripheral marker; on the contrary, if the nystagmus is towards the hypotropic eye (called DOWNHILL), this could be considered a central marker [87].

### 4.4. Spontaneous Acquired Nystagmus in Brainstem Lesion

**Downbeat nystagmus** (DBN) is the most common form of acquired central nystagmus. Usually, the fast phase beats in a downward direction, and it increases when looking down and in lateral gaze with associated static and dynamic postural instability and, sometimes, oscillopsia [88,89]. Although DBN is mostly due to a cerebellar disease (especially flocculus lesion [4,88]), some individual cases of DBN were described in cases of paramedian lesions of the medulla oblongata [20,21,88,89] or from vascular pathologies such as dolichoectasia of the vertebrobasilar circulation and compression of the vertebral artery [90,91]. **Upbeat nystagmus** (UBN) is a type of central vestibular nystagmus that is less common than DBN. UBN usually increases with upward gaze, and it is associated with impaired upward pursuit; usually, it does not increase on lateral gaze and may evolve into downbeat nystagmus with convergence [4,22]. UBN can be observed only during the acute stage of illness and resolves earlier than other ocular motor abnormalities [92]. Another very peculiar feature of UBN is its possible spontaneous transformation, in the course of the disease, into a hemi-seesaw, horizontal, and above all DBN [93]. Although the exact mechanisms remain to be elucidated, it is believed that critical structures for UBN are located at the midline in the lower medulla (ventral tegmental tract, the superior vestibular nucleus, the nucleus of Roller, and the solitary nucleus) [20,89]. UBN occurs as a result of lesions in various locations and is often seen associated with pontomedullary and pontomesencephalic lesions [13,22,92], and in contrast to downbeat nystagmus, lesions of the paramedian brainstem are frequent in upbeat nystagmus [15,93,94,95]; usually, UBN is associated with other brainstem signs, such as ataxia or dysarthria [94]. **Torsional nystagmus** (TN) often occurs as a consequence of pontomedullary junction lesions [96]. Primarily, TN arises from lesions in neural pathways involved in controlling torsional movements: the medulla (vestibular nuclei) and midbrain (INC and riMLF) [96]. Disruption of the otolithic–ocular pathways causes unbalanced torsional inputs to the ocular motor nuclei, leading to rotation of the eyes around the visual axis. TN, especially if isolated, suggests brainstem or vestibular nucleus lesions (e.g., Wallenberg syndrome [12,97], lesions of MLF and/or ICP [18,19]) and often coexists with vertical or gaze-evoked components [96]. **Gaze-evoked nystagmus (GEN)** arises from an impairment of the cerebellar-brainstem-mediated horizontal or vertical gaze-holding mechanism [35], inducing an inability to hold the eyes in a position of eccentric fixation. Horizontal GEN is a central sign with an excellent specificity [32] and, when combined with testing for other oculomotor signs (SD, HIT), is very useful in order to diagnose a central vestibular lesion in patients with acute vertigo [41,44,45,46,47,48,52]. Unlike end-point nystagmus (a benign finding, conjugate, in both right and left directions of gaze, transient, and more prominent with age), GEN is sustained, larger in amplitude, possibly asymmetric, and is often associated with DBN. GEN is frequently correlated to cerebellar diseases, but it is a common finding in cases of medial medullary infarction [15] and in unilateral lesions of NPH [16], and it was also described in pontine lesions [4]. On the other hand, a vertical GEN is because of an involvement of the pontine areas connected by the medial longitudinal fascicule (MLF) to the midbrain area involved with vertical gaze [25]. Frequently associated with GEN, **rebound nystagmus** (RN) could be observed, particularly in cerebellar disease. RN is a transient centripetal nystagmus that appears on returning to straight-ahead gaze after prolonged eccentric gaze. The slow phase is in the direction of prior eccentric gaze. RN could be considered a variant of GEN, as the reversal of the beating direction of GEN after gaze is shifted from an eccentric position to the straight-ahead eye position [98]. RN was described in subjects with lesions of NPI and the medial vestibular nucleus after the ability to eccentrically fixate (initially lost) was partially recovered [99]. Other rare types of nystagmus encountered in brainstem lesions are **seesaw nystagmus:** seesaw nystagmus refers to mixed torsional–vertical nystagmus with one eye moving upward and incyclorotating, and the other eye moving downward and excyclorotating. During the next half cycle, the vertical and torsional movements reverse. **Hemi-seesaw nystagmus** is a jerky nystagmus in which the slow phase corresponds to one-half cycle and the quick phase to the other [4,100], and it can be a finding of a medial [93] or lateral [100] medullary syndrome and midbrain lesion [101]. **Periodic alternating nystagmus** is a horizontal, conjugate, jerk nystagmus that periodically alternates its direction of fast phase. It is a very rare finding in brainstem lesions, and it was described occasionally in lesions involving the area of the central–dorsal medulla, including the bilateral NPH [102]. **Acquired pendular nystagmus** is characterized by slow-phase eye movements in the horizontal, vertical, and torsional planes, resulting in nearly sinusoidal movement. This nystagmus, commonly associated with oculo-palatal tremor, is related to an abnormal activity in central gaze-holding structures (NPI, medial vestibular nuclei, and INC) [103], especially induced by multiple sclerosis [26,104] and, less frequently, by other diseases affecting the brainstem [105]. **Convergence retraction nystagmus** is the most characteristic clinical sign of dorsal lesions of the rostral midbrain: this nystagmus consists of rapid inward–outward movement of the eye, followed by a slow return movement of protrusion and divergence when the patient is asked to look upward. It may be associated with upward gaze palsy in Parinaud syndrome [4,106]. **Dissociated nystagmus** is characterized by a difference in the direction, extent, or periodicity of the ocular movement between the two eyes. The most common cause of a dissociated nystagmus is a lesion of the MLF with a consequent INO (see Section 2.2). In the context of the so-called “nystagmoid movements”, we must remember the **ocular flutter,** characterized by rapid, short-duration ocular oscillations on the horizontal plane that appear spontaneously during fixation and end abruptly. Ocular flutter is often triggered by blinking or voluntary eye movements. This condition reflects dysfunction in saccade-generating brainstem circuits, especially the PPRF and cerebellar fastigial nucleus [107]. **Ocular bobbing** is a vertical nystagmoid movement characterized by rapid downward eye jerks followed by slow upward drifts and is typically associated with lesions in the PPRF in the ventral pons, where omnipause neurons disrupt saccadic inhibition, allowing uncontrolled vertical saccades [108].

Table 3 schematically summarizes the main characteristics of some of the disorders described in the different sections of the manuscript.

## 5. Conclusions

In addition to its several physiological functions of vital importance, the brainstem represents a structure of prominent importance for the generation and control of eye movements (saccades, smooth pursuit, the vestibulo-ocular reflex (VOR), vergence, and gaze holding). The clinical examination of the different eye movements and nystagmus allows, in most cases, topographic–anatomic diagnosis of central vestibular lesions due to brainstem abnormalities both in degenerative and progressive diseases and in acute (vascular) disorders. As a general rule, isolated impairments of vertical eye movements (in addition to pupillary and eyelid abnormalities) are indicative of midbrain damage, while isolated impairments of horizontal eye movements are more typical of a lesion in the pons. However, oculomotor abnormalities arising from brainstem disease constitute an extremely complex challenge for the neurotologist by virtue of the remarkable diversity of causes (vascular, traumatic, degenerative, neoplastic); of their variable severity; of their clinical course (acute, fluctuating, or progressive); and finally, by virtue of the concomitant involvement of other central structures (mainly the cerebellum). For these reasons, eye movement disorders and nystagmus of brainstem origin are frequently associated not only with vertigo and imbalance but also with a wide range of other neurological signs. Consequently, an accurate characterization of the eye movement abnormalities and a complete bedside and instrumental examination of the vestibular system associated with the fundamental contribution of the brain imaging (especially MRI) represents a fundamental moment in the diagnosis of central vestibular syndromes, being also able to identify and localize, at least in a good number of cases, the role of each unit and/or neural circuit in the genesis of the oculomotor lesion.

## Figures and Tables

**Table 1:** The examination of the ocular motor and the vestibular systems. VOR: vestibulo-ocular reflex.

Type of Examination	Search for
Head posture	Head tilt
Eye movements *Position of the eyes* *Straight ahead, look to the right, left, upward, and downward, cover test*	Primary misalignment, Spontaneous nystagmus Gaze function End-point nystagmus
Smooth pursuit	Saccadic,
	Reduction in gain
Saccades	Latency, velocity, accuracy
VOR functionality *Clinical head impulse test*	Presence of corrective saccades
Visual fixation suppression of the VOR	No suppression of VOR *(mainly occur in cerebellar diseases)*

**Table 2:** Summary of the main symptoms and signs of brainstem disorders and the relative involved structures.

Symptoms and Signs	Structures Involved
Vertigo, nystagmus	Vestibular nuclei
Tinnitus, hearing loss	Auditory nerve, cochlear nuclei
Gait and limb ataxia	Ventral spinocerebellar tract, middle cerebellar peduncle
Dysphagia, dysarthria	Vagal nuclei and nerve
Facial hemianesthesia	Fifth nerve and nucleus
Facial paralysis	Seventh nerve
Crossed hemisensory loss	Spinothalamic tract
Horner’s syndrome (ptosis, miosis, facial anhidrosis)	Descending sympathetic tract

**Table 3:** A brief summary of the main brainstem lesion/syndrome causing oculomotor disorders and pathological nystagmus. PICA: posterior inferior cerebellar artery. NPH: nucleus propositus hypoglossi. GEN: gaze-evoked nystagmus. HSN: head-shaking nystagmus. PPRF: paramedian pontine reticular formation. MLF: medial longitudinal fasciculus. riMLF: rostral interstitial nucleus of the medial longitudinal fasciculus. HIT: head impulse test.

Lesion/Syndrome	Primary Structure(s) Involved	Key Oculomotor and Nystagmus Characteristics
**Wallenberg Syndrome**	Lateral medulla (PICA territory)	**Nystagmus:** Spontaneous horizontal–torsional (fast phase beating away from the lesion). **Saccades:** Hypermetric (ipsilesional), hypometric (controlesional). **Associated sign:** ocular tilt reaction ipsilesional.
**Medial Medullary Infarction**	Nucleus propositus hypoglossi (NPH)	**Nystagmus:** Ipsilateral horizontal (sometimes *upbeating*); gaze-evoked nystagmus (GEN) more intense when looking toward the affected side. **Smooth pursuit:** Central pattern of head-shaking nystagmus (HSN).
**Pons Lesions (Horizontal Gaze)**	PPRF (paramedian pontine reticular formation)	**Saccades: Isolated horizontal saccadic palsy**. **Smooth pursuit:** Severely impaired or absent.
**Internuclear Ophthalmoplegia**	Medial longitudinal fasciculus (MLF)	**Unilateral:** Impaired adduction (ipsilateral eye) and abducting nystagmus (contralateral eye). **Bilateral:** Bilateral adduction latency/impairment and bilateral abducting nystagmus. **Convergence** is typically spared.
**“One and a Half” Syndrome**	PPRF + ipsilateral MLF	**Horizontal movements: Loss of all horizontal movements**, except for abduction in the eye contralateral to the lesion.
**Midbrain Lesions (Vertical Gaze)**	riMLF and interstitial nucleus of Cajal	**Saccades: Isolated vertical saccadic palsy**. **Nystagmus:** Possible isolated vertical GEN.
**Central Positional Nystagmus**	Vestibulo-cerebellar pathways (e.g., nodule/uvula)	**Latency/Fatigue:** Absent/minimal latency and **non-fatigable (persistent)**. **Direction:** Often pure **downbeat** or **apogeotropic bidirectional horizontal**.
**Isolated Vestibular Nuclei Infarction**	Vestibular nuclei	**Nystagmus:** Spontaneous torsional–horizontal, beating away from the side of the lesion; direction-changing GEN. **HIT:** May be **positive** (an atypical finding for a central lesion).

## Data Availability

No new data were created or analyzed in this study. Data sharing is not applicable for this article.

## Supplementary Materials

The following supporting information can be downloaded at: https://www.mdpi.com/article/10.3390/audiolres15060150/s1, Video S1: Torsional spontaneous nystagmus in a patient with multiple sclerosis involving the inferior cerebellar peduncle. Video S2: Patient suffering from positional vertigo. Bedside examination: Apogeotropic bidirectional persistent positional nystagmus refractory to repeated repositioning maneuvers.

## Abbreviations

The following abbreviations are used in this manuscript:

VOR	Vestibulo-ocular reflex
NPH	Nucleus propositus Hypoglossi
NR	Nucleus of Roller
OTR	Ocular tilt reaction
PPRF	Paramedian pontine reticular formation
GEN	Gaze-evoked nystagmus
HSN	Head-shaking nystagmus
SP	Smooth pursuit
MLF	Medial longitudinal fascicle
INO	Internuclear ophthalmoplegia
SD	Skew deviation
riMLF	rostral interstitial nucleus of the medial longitudinal fasciculus
PICA	Posterior inferior cerebellar artery
AICA	Anterior inferior cerebellar artery
ICP	Inferior cerebellar peduncle
MRI	Magnetic resonance imaging
HIT	Head impulse test
CNP	Central positional nystagmus
PDN	Positional downbeating nystagmus
BPPV	Benign paroxysmal positional vertigo
DBN	Downbeat nystagmus
UBN	Upbeat nystagmus
TN	Torsional nystagmus
